# Systematic Pathway Screening via Integrated Machine Learning Identifies FOXO‐Mediated Transcription Signature for Robust Immunotherapy Response Prediction in Non–Small Cell Lung Cancer

**DOI:** 10.1155/humu/8690530

**Published:** 2026-01-19

**Authors:** Shuqi Wu, Chenxi Deng, Chaofan Fan, Qiheng Liang, Lingxuan Zhu, Weiming Mou, Hongsen Huang, Keren Wu, Yizhang Li, Gengwen Deng, Liling Xu, Jiarui Xie, Chenglin Hong, Yuhang Deng, Xingjian Li, Changda Wu, Tao Yang, Peng Luo, Hank Z. H. Wong, Aimin Jiang, Anqi Lin, Xin Chen

**Affiliations:** ^1^ Department of Oncology, Zhujiang Hospital, Southern Medical University, Guangzhou, Guangdong, China, fimmu.com; ^2^ The Second School of Clinical Medicine, Southern Medical University, Guangzhou, Guangdong, China, fimmu.com; ^3^ Department of Pulmonary and Critical Care Medicine, Zhujiang Hospital, Southern Medical University, Guangzhou, Guangdong, China, fimmu.com; ^4^ School of Stomatology, Southern Medical University, Guangzhou, Guangdong, China, fimmu.com; ^5^ Department of Urology, Shanghai General Hospital, Shanghai Jiao Tong University School of Medicine, Shanghai, China, shsmu.edu.cn; ^6^ The First School of Clinical Medicine, Southern Medical University, Guangzhou, Guangdong, China, fimmu.com; ^7^ Department of Medical Oncology, National Cancer Center/National Clinical Research Center for Cancer/Cancer Hospital, Chinese Academy of Medical Sciences and Peking Union Medical College, Beijing, China, cacms.ac.cn; ^8^ Li Ka Shing Faculty of Medicine, The University of Hong Kong, Hong Kong SAR, China, hku.hk; ^9^ Department of Urology, Changhai Hospital, Naval Medical University (Second Military Medical University), Shanghai, China, chhospital.com.cn

**Keywords:** FOXO-mediated transcription pathway, immune checkpoints, immune infiltration, immunotherapy, non–small cell lung cancer

## Abstract

**Background:**

Non–small cell lung cancer (NSCLC), accounting for 80% of lung cancer cases, remains a leading cause of cancer‐related mortality globally. While immune checkpoint inhibitors (ICIs) have improved outcomes, their efficacy is limited to a subset of patients, necessitating robust biomarkers for personalized immunotherapy response prediction.

**Methods:**

We integrated transcriptomic data from 584 NSCLC patients across four cohorts treated with ICIs. Using 12,025 pathways from MSigDB, we applied 101 machine learning algorithm combinations (e.g., random survival forest [RSF], least absolute shrinkage and selection operator [Lasso], and Cox proportional hazards model with component‐wise likelihood‐based boosting [CoxBoost]) to identify prognostic signatures. OAK was used as the training set and Ravi, Jung, and Poplar as the validation set. The optimal pathway and algorithm combination was determined based on the average concordance index (*C*‐index) ranking in the validation sets, and a predictive model was generated. Performance was assessed by *C*‐index, receiver operating characteristic (ROC) analysis, and survival analysis. Biological relevance was evaluated through gene set enrichment analysis (GSEA), immune infiltration profiling, and immunohistochemistry (IHC).

**Results:**

The FOXO‐mediated transcription pathway combined with Lasso‐RSF algorithms emerged as the top predictor. The derived FOXO‐related signature (FRS) stratified patients into high‐risk and low‐risk groups, with high‐risk patients showing significantly worse progression‐free survival (PFS) and overall survival (OS) across all cohorts (*p* < 0.05). FRS outperformed clinical variables and 43 published models in predictive accuracy. IHC confirmed elevated expression of FRS‐associated genes (PCK1, IGFBP1) in nonresponders. Immune profiling revealed enriched antitumor immunity in low‐FRS patients.

**Conclusion:**

FRS, a machine learning–derived pathway signature, robustly predicts immunotherapy response and survival in NSCLC. Its integration of FOXO‐mediated immune regulation offers a clinically translatable tool for precision oncology.

## 1. Introduction

Globally, lung cancer remains the foremost cause of cancer‐related mortality among both men and women, imposing a substantial burden on public health systems, particularly in developing countries [1–5]. The World Health Organization categorizes lung cancer into two primary groups [6], of which non–small cell lung cancer (NSCLC) accounts for over 80% of all lung cancer cases [7]. However, the 5‐year survival rate is only 15% [8].

The treatment of lung cancer is challenging due to severe symptoms and a poor prognosis. Although various treatment strategies, including surgery, chemotherapy, radiotherapy, and targeted therapy [9], can be employed for patients with NSCLC, the high metastasis rate and poor survival of this disease urgently require the development of more effective treatment strategies and active control. Compared to chemotherapy, immunotherapy has fewer side effects and can extend patients′ survival while also improving their quality of life [10–15]. In recent years, the rapid development of tumor immunology has deepened our understanding of the immune system response in tumor tissues and has greatly contributed to the progress of immunotherapy. In NSCLC, the presence of immune cells within the cancer and antigens different from those in normal lung tissue enhances the immune activity in the lungs, suggesting that immunotherapy may have an important role in NSCLC [16].

Over the past decade, the use of immunotherapy strategies involving programmed death 1 (PD‐1)/programmed death ligand 1 (PD‐L1) immune checkpoints has transformed the treatment modalities and therapeutic landscape of NSCLC. Some patients with metastatic NSCLC have achieved durable response rates and long‐term survival [17]. However, the tumor microenvironment (TME) of NSCLC is affected by a variety of driver oncogenes (drivers) and exhibits significant heterogeneity, which leads to different sensitivities of patients to treatment with immune checkpoint inhibitors (ICIs) for PD‐L1/PD‐1 [18]. Consequently, there is a pressing need to identify dependable methodologies for anticipating the responsiveness of NSCLC patients to immunotherapy and for identifying patients who may derive benefit from ICI therapy, taking into account the financial implications, potential adverse effects, and the inevitable problem of drug resistance [19].

To date, more than 40 predictive biomarkers, including tumor mutation burden (TMB), PD‐L1, and microsatellite instability (MSI), have been evaluated. However, no single biomarker is effective enough to predict the effectiveness of ICIs. Cutting‐edge pathways are functional ensembles comprising a series of interacting molecules and signals that regulate biological processes such as tumor cell growth, proliferation, invasion, and metastasis in NSCLC. Due to the complex interactions between tumors, TMEs, and host immunity, a promising approach for predicting outcomes after ICI application in NSCLC may be to characterize multigene signaling pathways.

Machine learning, which can identify the significance of features and enhance the accuracy of predictive models, has been extensively employed for the construction of predictive models in NSCLC [20–22]. However, the majority of predictive models are often challenging to implement in clinical settings due to inadequate training and validation datasets, inappropriate machine learning methods, and a lack of suitable immunotherapy cohorts. We posit that machine learning–based feature marker extraction with cross‐cohort data validation represents a flexible and powerful approach to personalized NSCLC immunotherapy patient risk stratification and treatment selection.

In this study, we apply 101 algorithms with 12,025 pathways in combination to identify machine learning–derived feature pathway models (FOXO‐related signature [FRS] model) for the development and validation of risk‐stratified features in 584 NSCLC patients undergoing ICI therapy from four independent public datasets. The objective is to assess patient recurrence‐free survival and ICI treatment benefit. This work contributes to the optimization of precision treatment for NSCLC and further improvement of the clinical prognosis of NSCLC patients.

## 2. Materials and Methods

### 2.1. Collection of NSCLC Patient and Tumor Cell Line Cohorts

The study included 584 patients with NSCLC who had been treated with ICIs in four independent cohorts. These were (1) OAK [23] (NCT02008227), which included 344 patients; (2) Poplar [24] (NCT01903993), which included 95 patients; (3) Ravi [25], which included 118 patients; and (4) Jung [26], which included 27 patients. The cohorts provided survival information, and patients underwent RNA sequencing of tissues prior to immunotherapy. The RNA sequencing raw read counts were converted to transcripts per kilobase of exon model per million mapped reads (TPM) and subsequently converted to log‐2 (TPM + 1).

The metacohort was constructed by integrating data from three independent cohorts: Ravi, Jung, and Poplar. Batch effects were corrected using the ComBat algorithm from the sva package. The batch‐corrected expression data were then merged with clinical and survival information to create a unified metacohort.

### 2.2. Multipathway Integrated Machine Learning

We implemented a comprehensive machine learning framework to identify pathway‐based signatures predictive of immunotherapy response in NSCLC. Initially, we acquired 12,025 pathways from the MSigDB database, encompassing various functional collections including HALLMARK, REACTOME, KEGG, GOBP, GOCC, and GOMF [27]. For each pathway, we performed univariate Cox regression analysis with progression‐free survival (PFS) as the endpoint. Only genes significantly associated with PFS (*p* < 0.05) were retained as candidate features for subsequent machine learning modeling.

To ensure robust feature selection and model optimization, we employed a multialgorithm approach by integrating 10 distinct machine learning algorithms [28]: random survival forest (RSF), least absolute shrinkage and selection operator (Lasso), Cox proportional hazards model with component‐wise likelihood‐based boosting (CoxBoost), elastic net (Enet), gradient boosting machine (GBM), partial least squares regression for Cox models (plsRcox), ridge regression, stepwise Cox proportional hazards regression (StepCox), supervised principal components (SuperPC), and support vector machine for survival analysis (survival‐SVM). These algorithms were implemented individually and in various combinations, ultimately resulting in up to 101 different algorithmic approaches for model building.

For our model development strategy, the OAK cohort (*n* = 344) served as the training dataset, while Ravi (*n* = 118), Jung (*n* = 27), and Poplar (*n* = 95) cohorts were used as independent validation datasets. Each pathway–algorithm combination was evaluated by fitting the model on the training set and assessing its performance across all three validation datasets. Performance was primarily quantified using the concordance index (*C*‐index), which measures the model′s ability to correctly rank patient survival outcomes. To identify the optimal pathway–algorithm combination, we calculated the average *C*‐index across all validation datasets for each combination and ranked them accordingly. This comparison identified the most effective pathway–algorithm combination as our final predictive model. The model demonstrated superior prognostic performance and generalizability across multiple independent cohorts, making it a potential clinically applicable biomarker for immunotherapy response prediction in NSCLC.

### 2.3. Immune‐Related Characteristics of FRS

Algorithms such as the xCell algorithm [29], the ESTIMATE algorithm [30], and the CIBERSORT algorithm [31] were employed to assess the intratumoral immune infiltrating cell profile [32]. Additionally, the expression of 25 key immune checkpoint–related genes, including members of the B7‐CD28 and TNF receptor superfamilies, was directly evaluated to investigate the immune‐regulatory landscape [33–38].

### 2.4. Differential Expression Analysis and Gene Set Enrichment Analysis (GSEA)

Differential expression analysis was performed using the limma package [39] to identify genes with significant changes across experimental groups. Differentially expressed genes were identified based on a false discovery rate (FDR) threshold of < 0.05 and an absolute log2 fold change (|logFC|) > 0.5. A total of 12,502 gene sets were obtained from the MSigDB database using the GSEA method [40]. The log2 fold change (logFC) values of differentially expressed genes were used to rank all genes in descending order. The ranked gene list was subjected to the clusterProfiler package [41, 42] to perform GSEA. Gene sets with a *p* value of less than 0.05 were considered significantly enriched.

### 2.5. Immunohistochemistry (IHC) Staining

Tumor tissues of 15 NSCLC samples from the in‐house dataset were further collected for IHC staining. Patient selection and sample collection were approved by the Medical Ethics Committee of Zhujiang Hospital (Approval Number 2022‐KY‐208‐01). Briefly, tissue sections were placed in citric acid antigen repair buffer (pH 6.0) in a microwave oven for antigen retrieval. Slices were placed in a 3% hydrogen peroxide solution to block endogenous peroxidase. 3% bovine serum albumin (BSA) was used as a blocking reagent. Primary antibodies against IGFBP1 (31025, CST) and PCK1 (ab133603, Abcam) were diluted in blocking reagent and incubated overnight. The sections were then incubated with a horseradish peroxidase–conjugated secondary antibody (1:200, GB23303, Servicebio). A 3‐3 ^′^‐diaminobenzidine staining kit (G1212, Servicebio) was used for color development, and hematoxylin was used for counterstaining cell nuclei.

Following immunostaining, the tissue sections were digitally scanned and analyzed. For each sample, three representative fields of view (FOVs) were randomly selected at the same magnification (40×) within the tumor regions. The digital images of these FOVs were then exported and further processed using the IHC Profiler plugin in ImageJ software (Version 1.54g, National Institutes of Health, United States). The plugin automatically quantified the immunoreactivity by measuring percentage of positively stained cells and staining intensity. For each FOV, a histochemical score (*H*‐score) was calculated using the formula: *H*‐score = ∑(percentage of cells in each intensity category × corresponding intensity score). The final *H*‐score for each sample was determined by averaging the *H*‐scores from the three selected FOVs.

### 2.6. Statistical Analysis

The timeROC package [43] was employed to plot the receiver operating characteristic (ROC) curves, which measured the predictive performance of the FRS model in PFS. The survival package [44] was used to perform univariate and multivariate Cox regression models. The ggplot2 package [45] was used to perform data visualization and present statistical results. For nonnormally distributed variables, significant quantitative differences between and among groups were determined by the Wilcoxon test; for categorical variables, significant differences were determined using the chi‐square test. All statistical tests were conducted with a two‐sided hypothesis. A *p* value of less than 0.05 was considered statistically significant. The software used in the analyses was run on the R (4.1.2) platform.

## 3. Results

### 3.1. Comprehensive Construction of Prognostic Model

**Figure 1:**
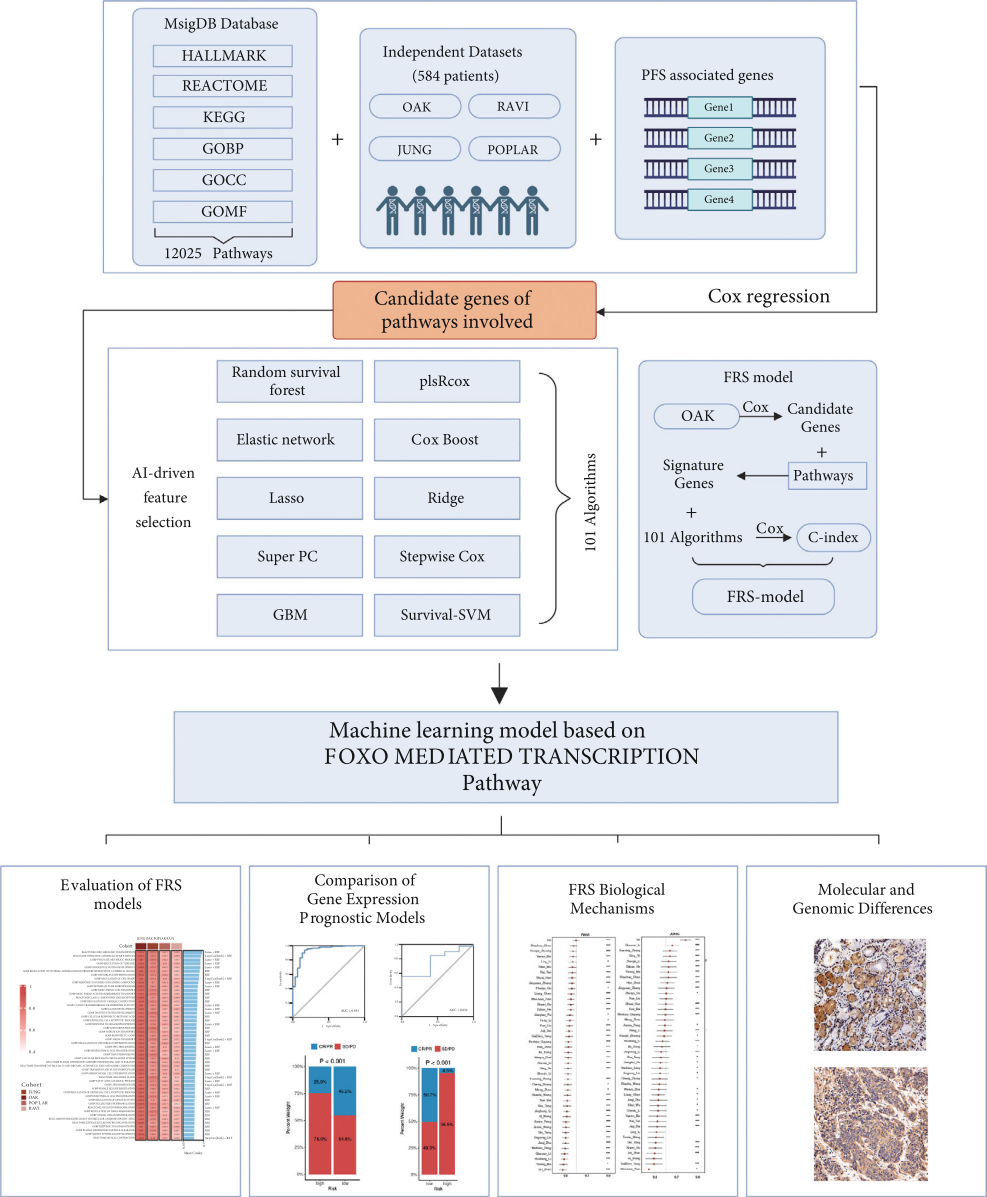
The computational framework for establishing the FRS signature. This study included 584 non–small cell lung cancer (NSCLC) patients, divided into four independent cohorts, who were treated with immune checkpoint inhibitors. Univariate Cox regression analysis was used to screen for genes affecting prognosis. A total of 12,025 pathways were downloaded from the MSigDB database to identify genes significantly associated with progression‐free survival (PFS) in NSCLC patients treated with anti‐PD‐1/PD‐L1 therapy. A combination of 10 machine learning algorithms was evaluated based on 10‐fold cross‐validation to identify the algorithm combination with the highest concordance index (*C*‐index). Ultimately, the Lasso‐RSF algorithm combination, which demonstrated the best performance, was selected to construct the FOXO‐related signature (FRS) model. The relationship between FRS prognosis, tumor immune microenvironment and immunotherapy response was comprehensively investigated. Abbreviations: RSF, random survival forest; Lasso, least absolute shrinkage and selection operator; CoxBoost, Cox proportional hazards model with component‐wise likelihood‐based boosting; Enet, elastic net; GBM, gradient boosting machine; plsRcox, partial least squares regression for Cox models; StepCox, stepwise Cox proportional hazards regression; SuperPC, supervised principal components; survival‐SVM, support vector machine for survival analysis; *C*‐index, concordance index; FRS, FOXO‐related signature; KEGG, Kyoto Encyclopedia of Genes and Genomes; GOBP, Gene Ontology biological process; GOCC, Gene Ontology cellular component; GOMF, Gene Ontology molecular function.

A total of 584 patients from four cohorts treated with ICI were included in the study. In order to identify the key pathways that influence the prognosis of patients undergoing ICI treatment, up to 101 algorithm combinations were created for each pathway using 12,025 pathways obtained from the MSigDB database, which were incorporated into the integrated machine learning pipeline. OAK was used as the training set, and Ravi, Jung, and Poplar as the validation sets (Figure 1). The FOXO‐mediated transcription pathway and the Lasso‐RSF algorithm combination were identified as the optimal pathway and algorithm combinations based on the average *C*‐index ranking in the validation sets. This combination exhibited the highest average *C*‐index (*C*‐index = 0.6918) and demonstrated consistent performance across all validation sets (Figure 2a). Based on this optimal combination, we developed the FRS model. Subsequently, we used the FRS model to calculate the FRS score for each patient. Given the heterogeneity across different cohorts, the cutoff value for distinguishing high‐risk and low‐risk FRS groups was independently determined for each dataset using the surv_cutpoint function from the R package survminer (Version 0.5.0). This method identifies the FRS score threshold that maximizes the log‐rank statistic for PFS within each specific cohort, allowing for cohort‐specific optimization of risk stratification. Kaplan–Meier survival analysis demonstrated that patients in the high‐risk group delineated by the model exhibited a worse PFS than those in the low‐risk group in both the training set and the three validation sets (Figures 2b, 2c, 2d, and 2e, *p* < 0.05). The same trend was observed in the metacohort integrating all samples and overall survival (OS) cohorts (Figures 2f, 2g, 2h, and 2i, *p* < 0.05).

Figure 2FRS was developed and assessed in OAK and multiple datasets. (a) The *C*‐index of the top pathway–algorithm combinations is shown for the training and validation cohorts. The mean *C*‐index (right) ranks the models, with the FOXO pathway combined with Lasso + RSF selected for the FRS model. (b–i) Kaplan–Meier survival analysis of the FRS model for progression‐free survival (PFS) and overall survival (OS). Patients were stratified into high‐risk (red) and low‐risk (blue) groups. The high‐FRS group consistently showed significantly worse survival (log‐rank test, *p* < 0.05) across the OAK training set, Ravi, Jung, Poplar validation sets, and the metacohort. Abbreviations: RSF, random survival forest; Lasso, least absolute shrinkage and selection operator; CoxBoost, Cox proportional hazards model with component‐wise likelihood‐based boosting; Enet, elastic net; GBM, gradient boosting machine; plsRcox, partial least squares regression for Cox models; StepCox, stepwise Cox proportional hazards regression; SuperPC, supervised principal components; survival‐SVM, support vector machine for survival analysis; OS, overall survival.

**Figure:**
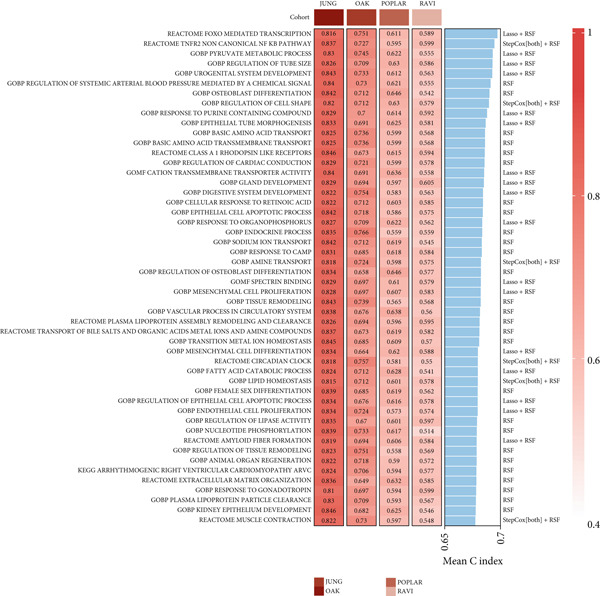
(a)

**Figure:**
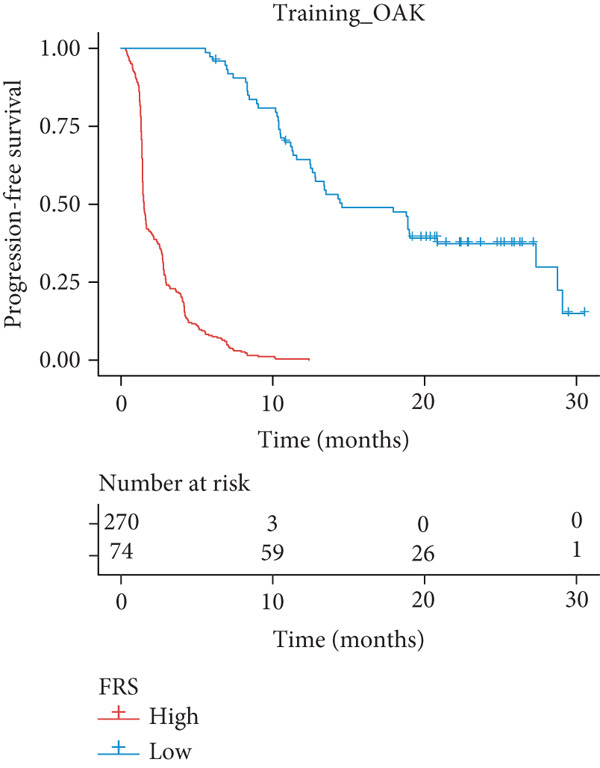
(b)

**Figure:**
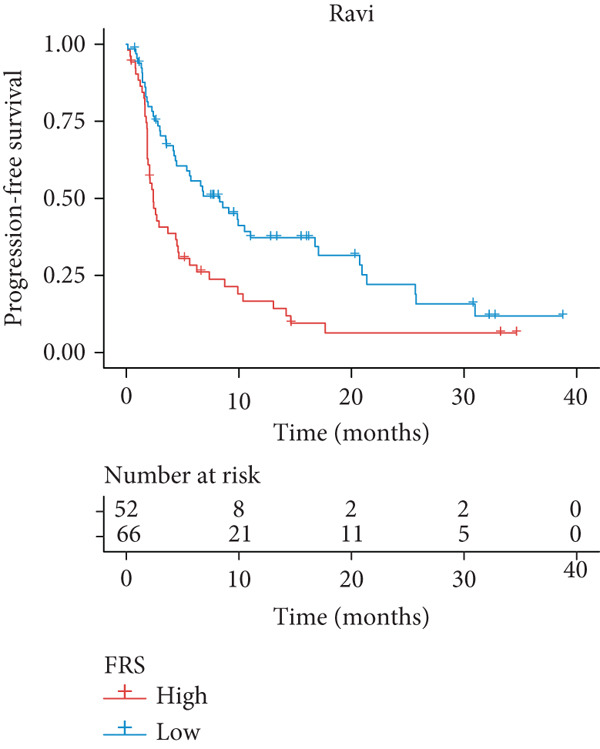
(c)

**Figure:**
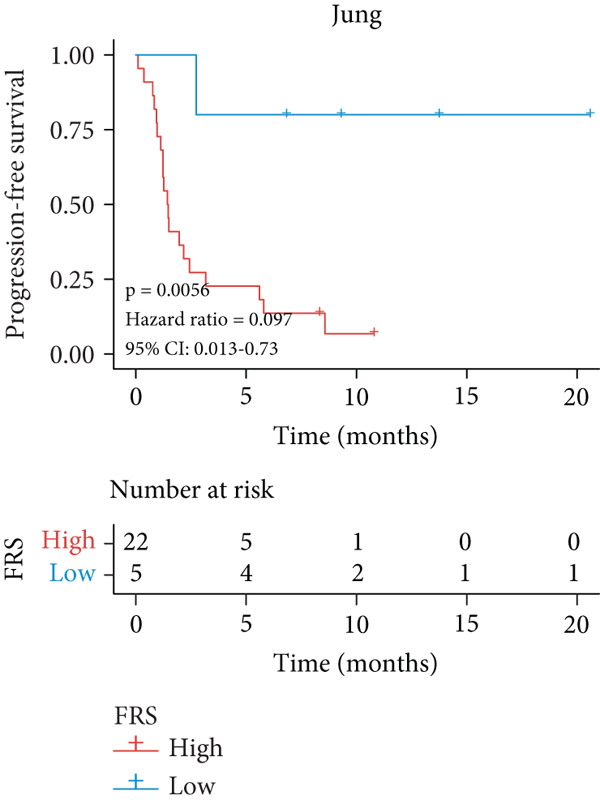
(d)

**Figure:**
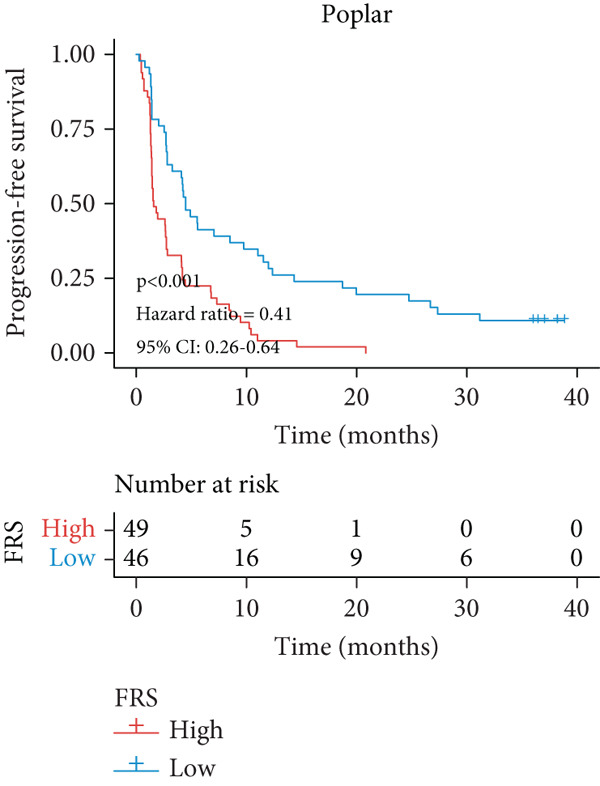
(e)

**Figure:**
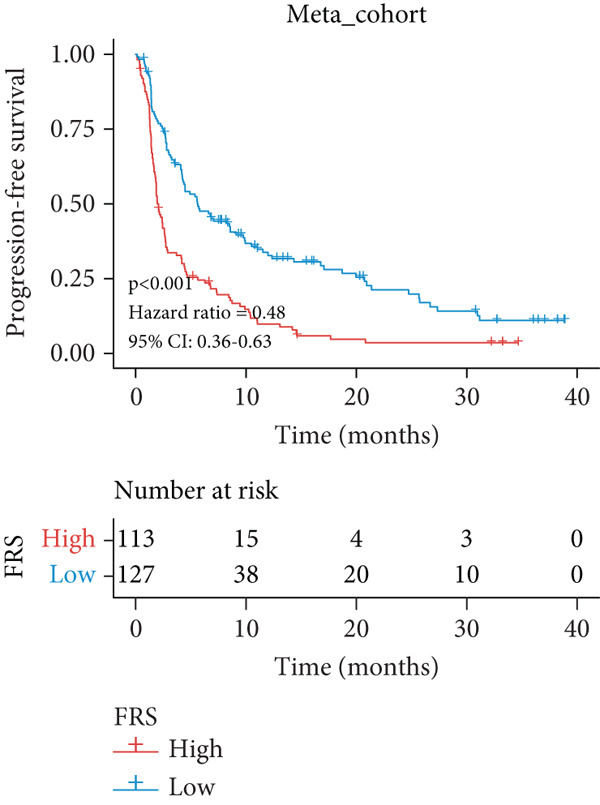
(f)

**Figure:**
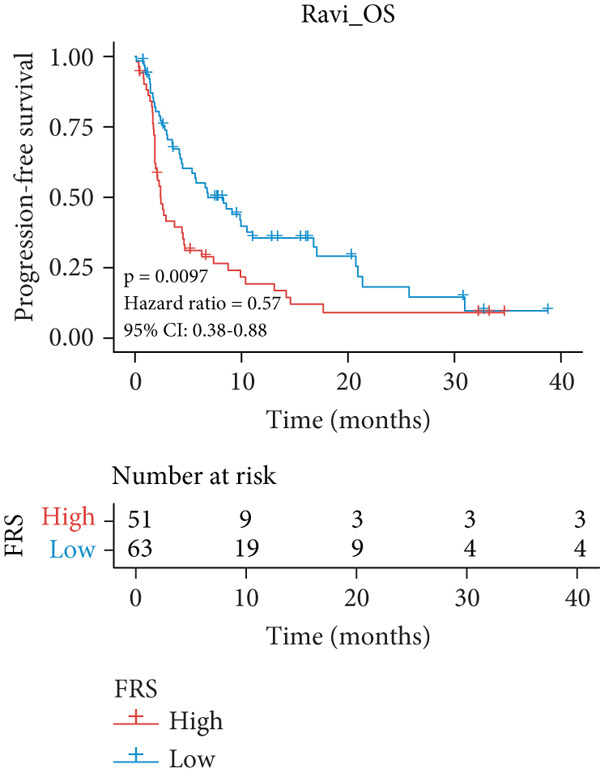
(g)

**Figure:**
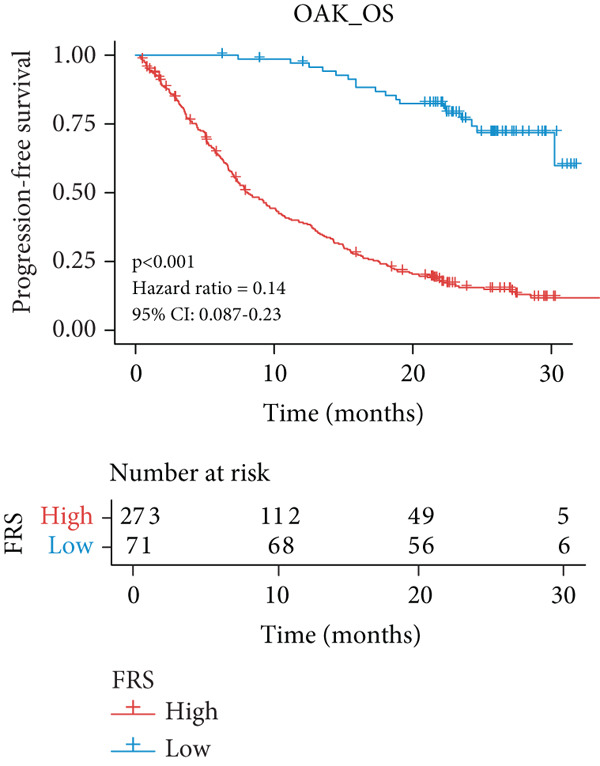
(h)

**Figure:**
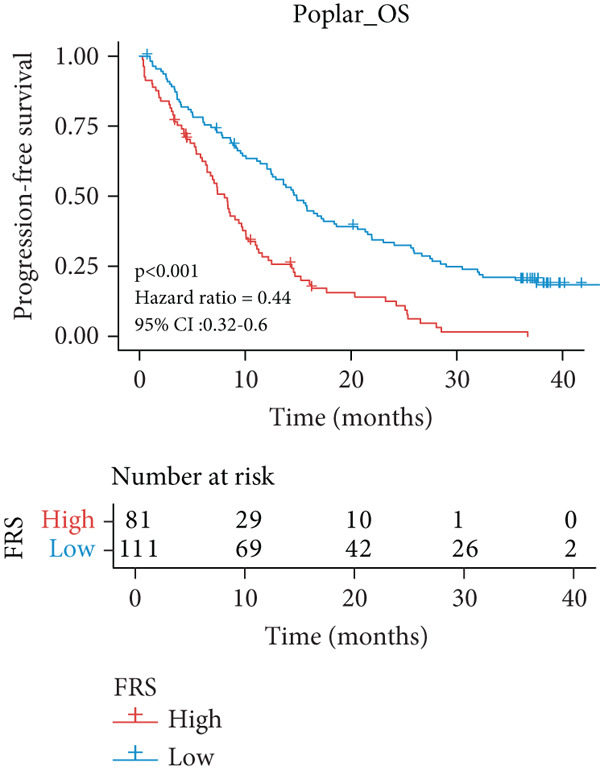
(i)

### 3.2. Evaluation of FRS Models

The predictive performance of FRS was determined by ROC analysis. Time‐dependent ROC curves, generated using the timeROC package, showed that the areas under the curve (AUCs) at 1, 3, and 5 years were 0.748, 0.974, and 0.991 in the OAK cohort (Figure 3a) and 0.665, 0.663, and 0.645 in the metacohort (Figure 3b), respectively. Additionally, we calculated standard ROC curves using the pROC package, which ignore the time factor and only reflect the overall predictive performance throughout the entire follow‐up period. The AUCs of the standard ROC curves were 0.933 in the OAK cohort (Figure 3c) and 0.652 in the metacohort (Figure 3d). Interestingly, we found that all the evidence suggested superior stability of FRS across multiple independent cohorts. Additionally, FRS demonstrated robust predictive performance in other types of tumors. In these studies, lower FRS scores were associated with patients having better OS (Figures 3e, 3f, and 3g).

Figure 3Evaluation of the FRS model. (a–d) Time‐dependent and standard receiver operating characteristic (ROC) curves for the FRS model in the OAK and metacohorts, with respective area under the curve (AUC) values. (e–g) Kaplan–Meier analysis showing the FRS effectively predicts overall survival (OS) in external cohorts of melanoma, urothelial carcinoma, and another NSCLC study. High‐risk patients are shown in red, low‐risk patients in blue. (h–k) Multivariate Cox analysis demonstrating that FRS is an independent prognostic factor for survival when compared with other clinical variables across four NSCLC cohorts. (l–n) Distribution of clinical response (CR/PR vs. SD/PD) by FRS risk group. Low‐risk patients showed significantly better response rates. Abbreviations: CR, complete response; PR, partial response; SD, stable disease; PD, progressive disease; AUC, area under the curve.

**Figure:**
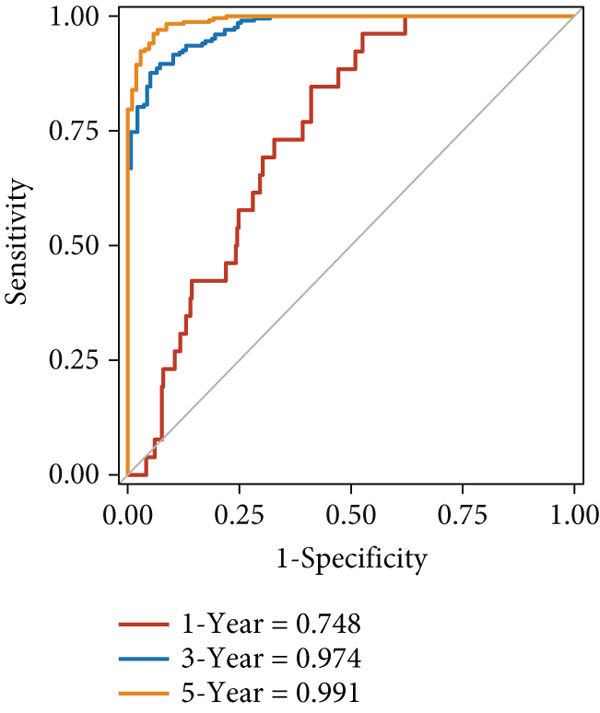
(a)

**Figure:**
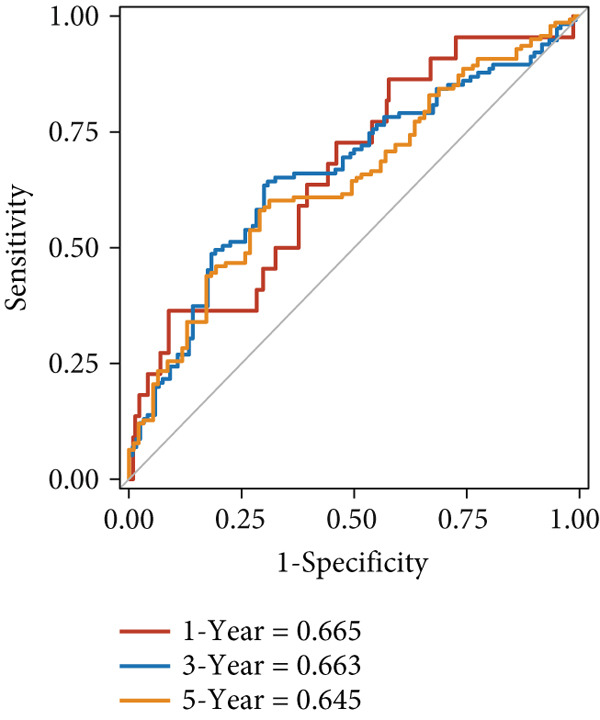
(b)

**Figure:**
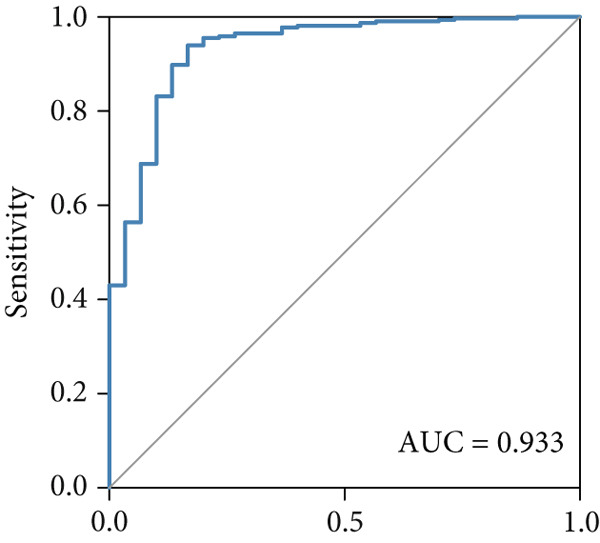
(c)

**Figure:**
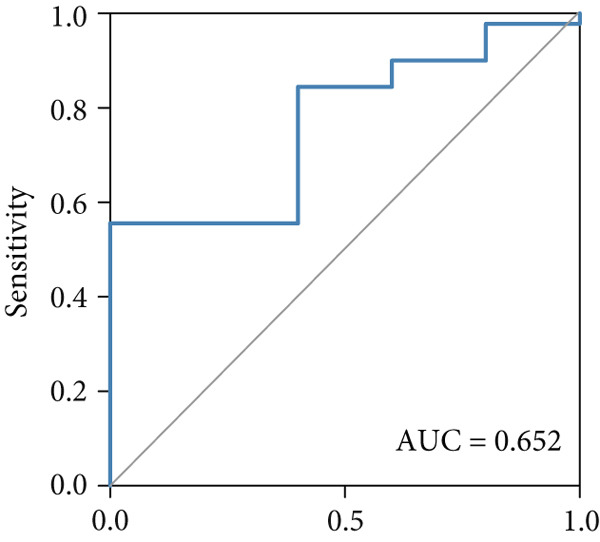
(d)

**Figure:**
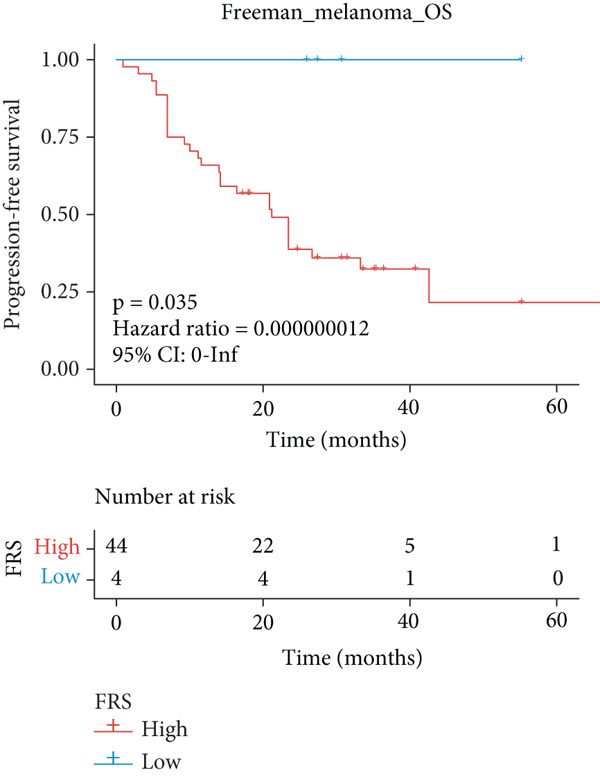
(e)

**Figure:**
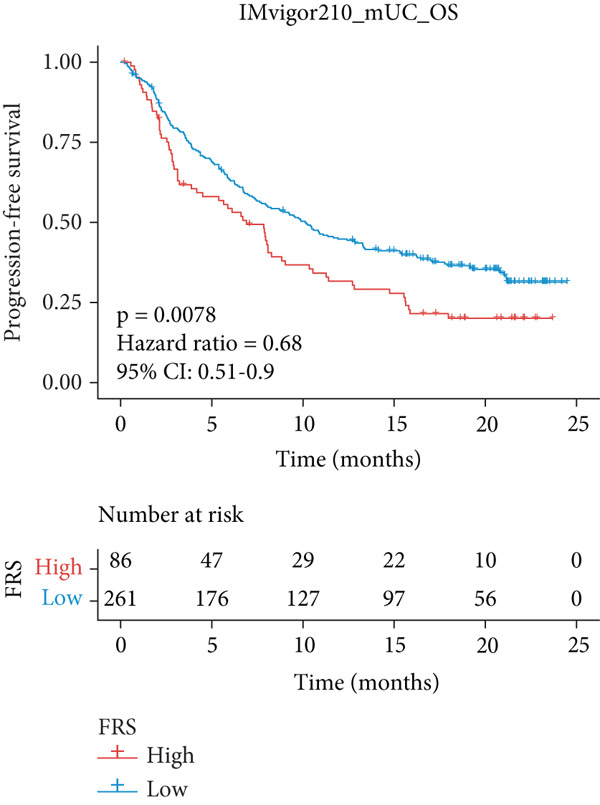
(f)

**Figure:**
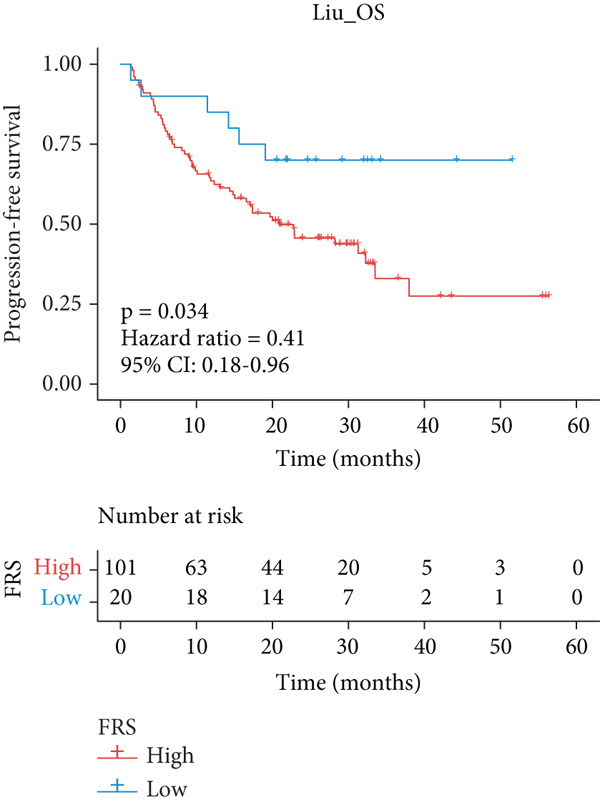
(g)

**Figure:**
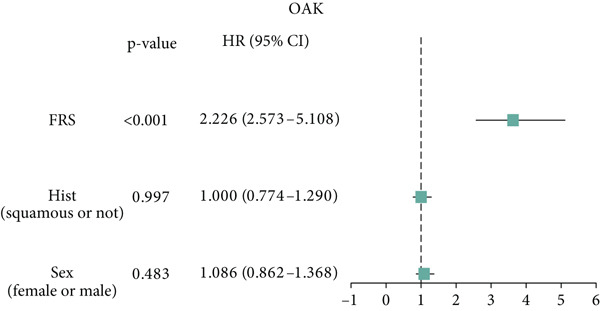
(h)

**Figure:**
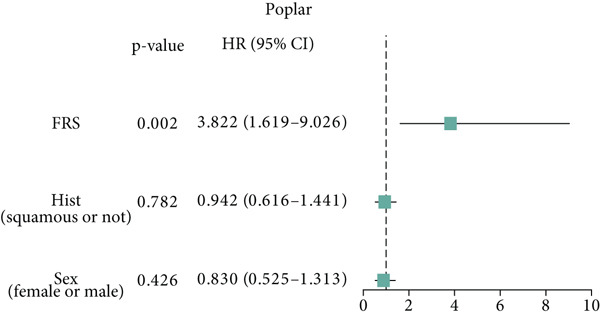
(i)

**Figure:**
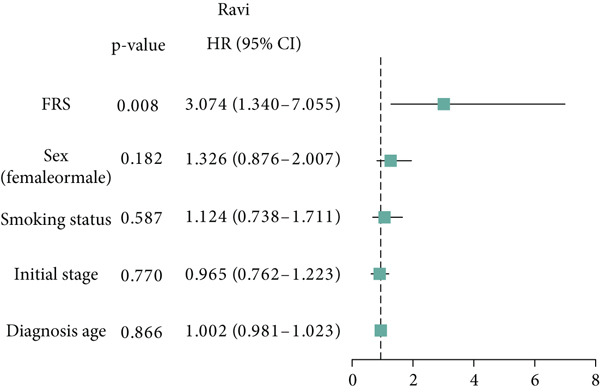
(j)

**Figure:**
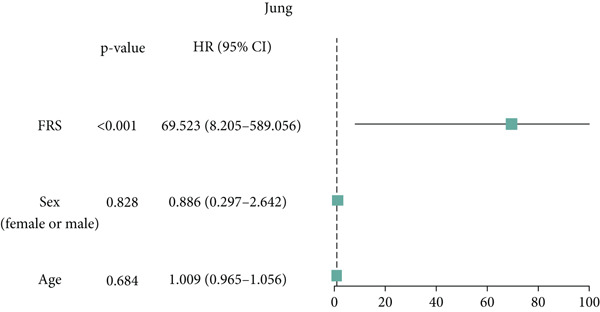
(k)

**Figure:**
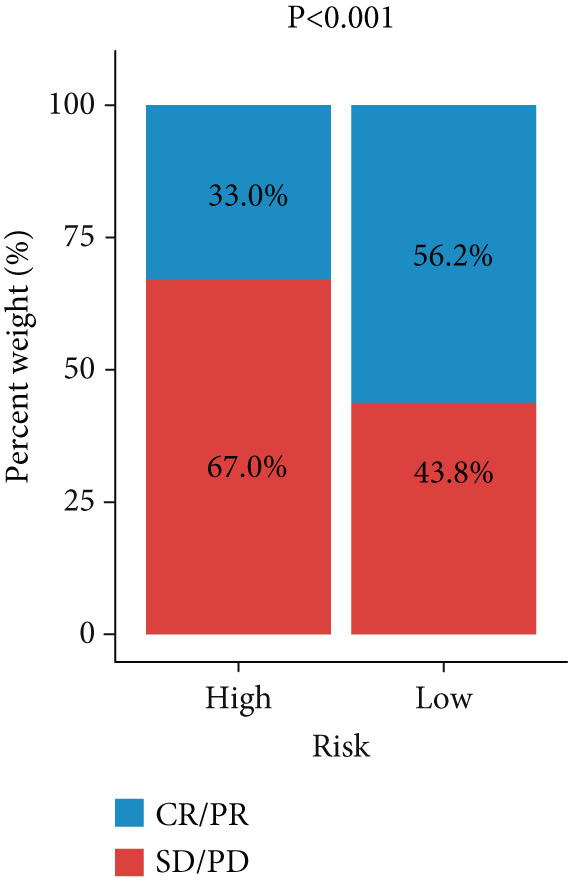
(l)

**Figure:**
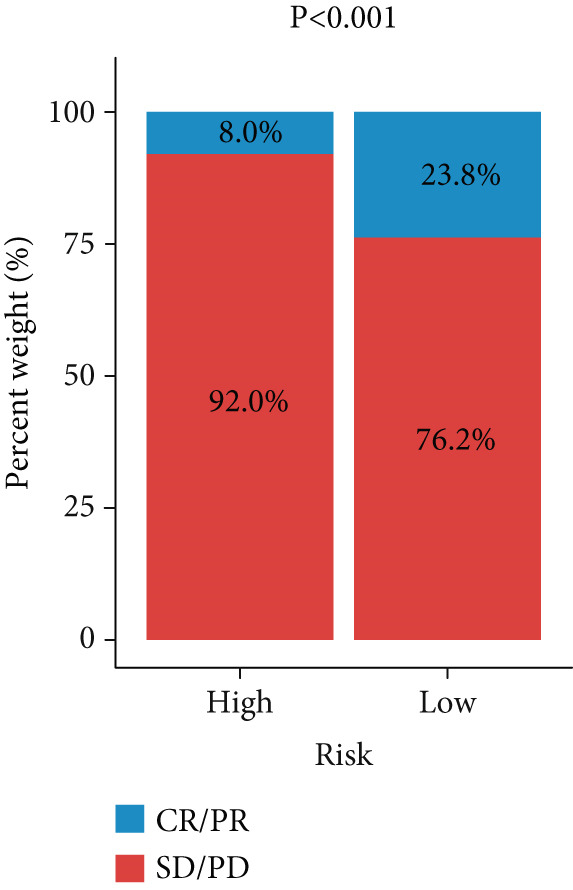
(m)

**Figure:**
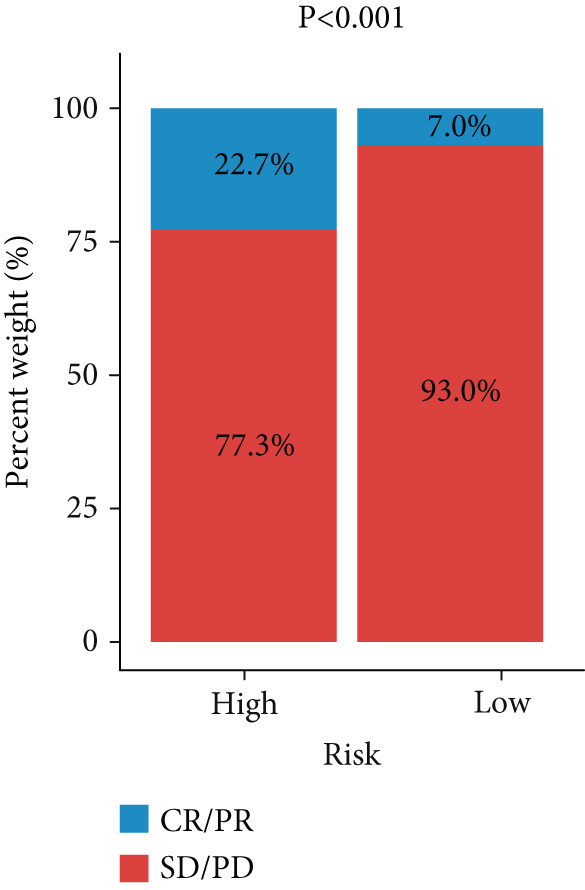
(n)

A number of clinical and molecular factors play a significant role in prognostic risk assessment and clinical decision‐making. Consequently, we conducted a comparison of the predictive accuracy of FRS with these variables by univariate Cox analyses. The results demonstrated that FRS exhibited significantly greater predictive accuracy than other variables, including age, gender, histology type, smoking status, and clinical stage (Figures 3h, 3i, 3j, and 3k). A comparison of the objective remission rates of high‐risk and low‐risk groups revealed that the low‐risk group exhibited superior objective remission rates in all cohorts. This finding was consistent with the survival analysis results, indicating that our FRS was effective in predicting the response to PD1/PDL1 inhibitors in NSCLC patients (Figures 3l, 3m, and 3n).

To further validate the FRS model in practice, we performed separate univariate Cox analyses of the genes in the model using the OAK cohort and other validation datasets. Notably, only **PCK1** and **IGFBP1** consistently demonstrated significant associations with inferior PFS outcomes (*p* < 0.05) across all four datasets (Figure 4a,b and Supporting Information 1: Figure S1A–C). Furthermore, to further validate the protein expression of PCK1 and IGFBP1 in samples with different clinical efficacy assessments, we performed IHC on samples from 8 NSCLC patients with a partial response (PR) and 7 with progressive disease (PD). Meanwhile, IHC images and *H*‐scores showed that PCK1 and IGFBP1 expression was significantly higher in PD samples (Figure 4c,d). This suggests that our model is promising for clinical application in NSCLC.

Figure 4Characterization of FRS models. (a) Univariate Cox analysis of FOXO pathway genes in the OAK cohort. (b) Venn diagram showing that PCK1 and IGFBP1 are the only two genes significantly associated with poor prognosis across all four cohorts. (c, d) Representative immunohistochemistry (IHC) images and quantification of protein expression (*H*‐score). Both IGFBP1 and PCK1 showed significantly higher expression in patients with progressive disease (PD) compared to those with a partial response (PR). Abbreviations: PR, partial response; PD, progressive disease.

**Figure:**
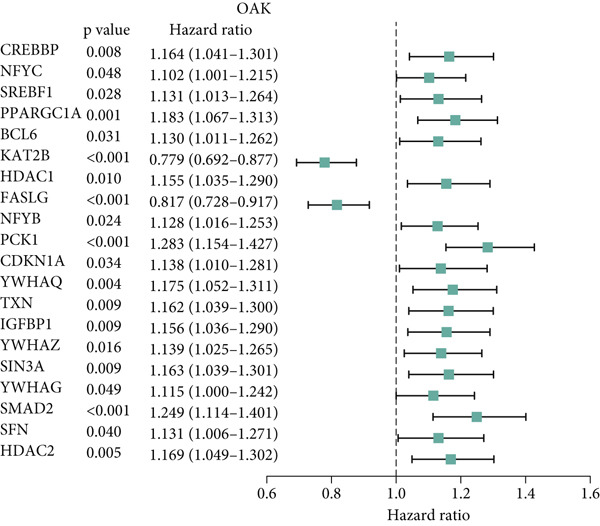
(a)

**Figure:**
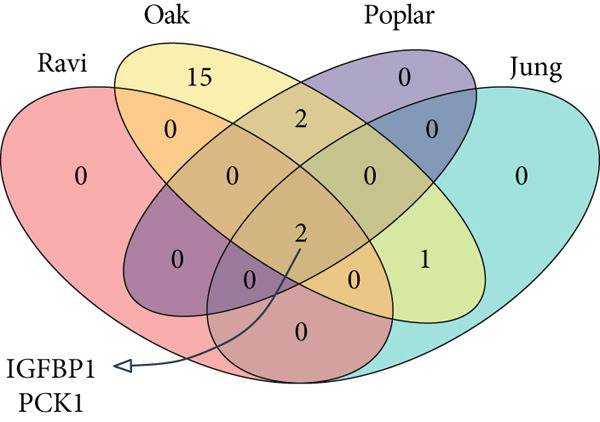
(b)

**Figure:**
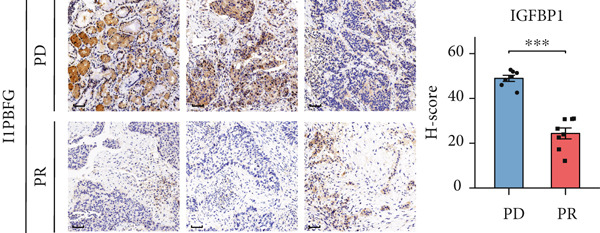
(c)

**Figure:**
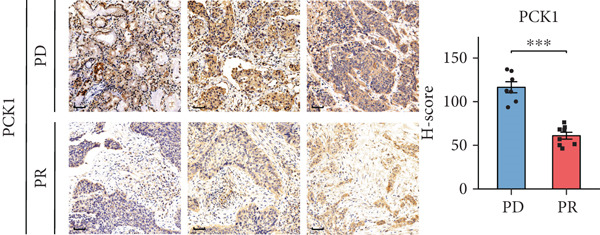
(d)

### 3.3. Comparison of Prognostic Feature Models Based on Gene Expression

The development and reporting of predictive models based on gene expression have been extensive. In order to comprehensively compare the predictive power of FRS models with that of other models, we collected 43 published models for predicting the prognosis of lung cancer patients receiving immunotherapy. These 43 models demonstrated a variety of important biological features, including neutrophil differentiation characteristics, cellular pyroptosis, epithelial–mesenchymal transition, inflammation, hypoxia, iron death, epigenetics, N6‐methyladenosine, TME, and endoplasmic reticulum stress (ERS). Univariate Cox regression was performed on all datasets for each model. The FRS model consistently demonstrated significant associations with inferior PFS outcomes in all cohorts (HR > 1, *p* < 0.05), highlighting its predictive concordance. In contrast, the other evaluated models exhibited inconsistent performance, with their prognostic significance not uniformly validated across the different cohorts (Figure 5a). Furthermore, the *C*‐index of FRS was compared with other models, and FRS demonstrated superior performance to almost all models in each dataset, while the predictive performance of all other models was unstable. It is noteworthy that the Wenhao Ouyang model has a higher *C*‐index than the FRS model in the Poplar dataset but performs poorly in the other datasets, indicating that the predictive performance and stability of the Wenhao Ouyang model are inferior to those of the FRS model (Figures 5b, 5c, 5d, and 5e). In summary, our FRS model was developed using two machine learning algorithms for dimensionality reduction, a process that enhances its potential for extrapolation and prediction.

Figure 5The FRS model demonstrates superior and more stable prognostic performance compared to 43 other published models. (a) Univariate Cox regression analyses conducted for each model across all datasets. The FRS model exhibited superior extrapolation and prediction potential. (b–e) A comparison of the *C*‐index of the FRS model with the other models. The FRS model demonstrated superior performance to almost all models in each dataset.

**Figure:**
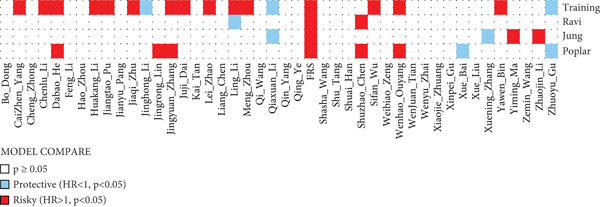
(a)

**Figure:**
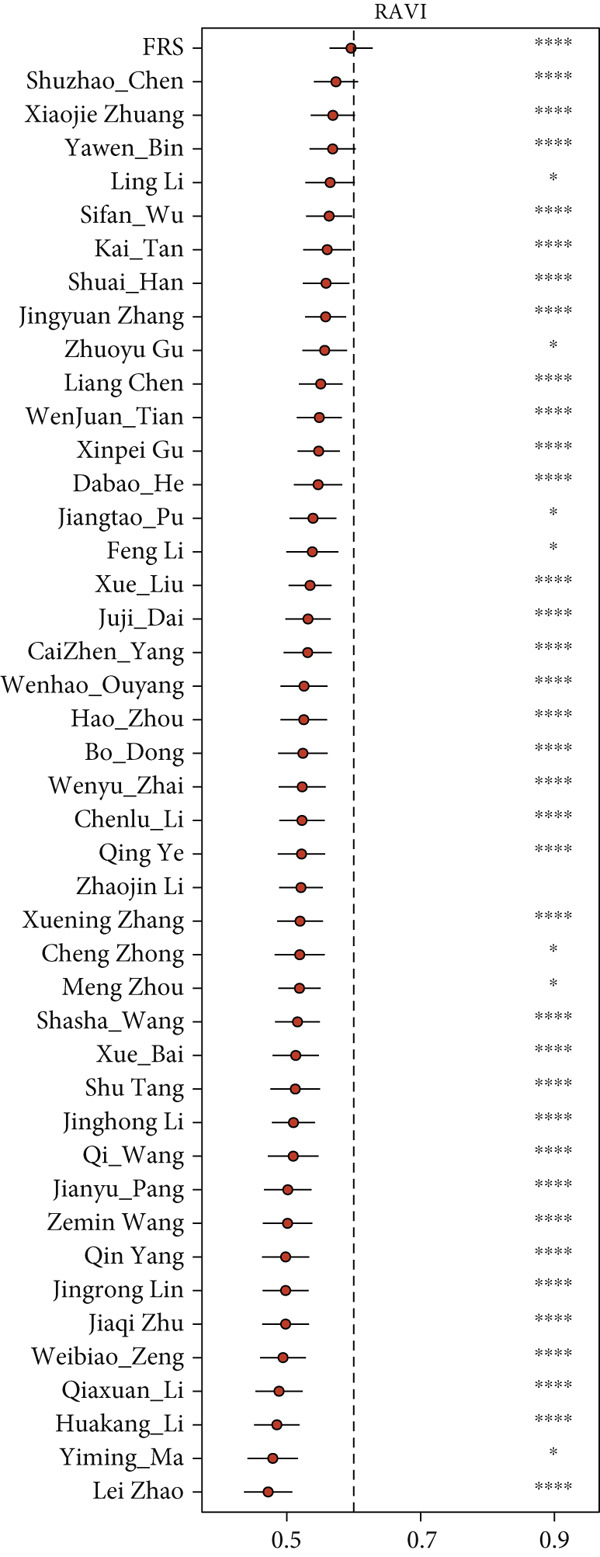
(b)

**Figure:**
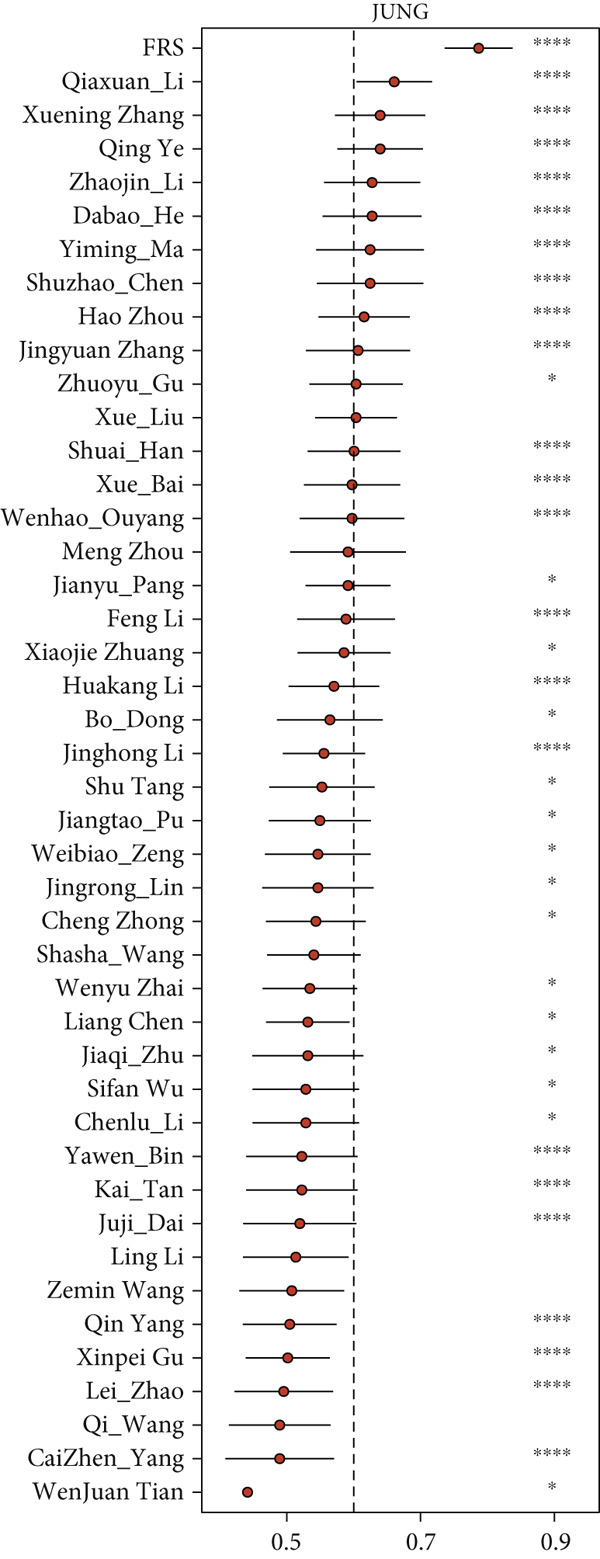
(c)

**Figure:**
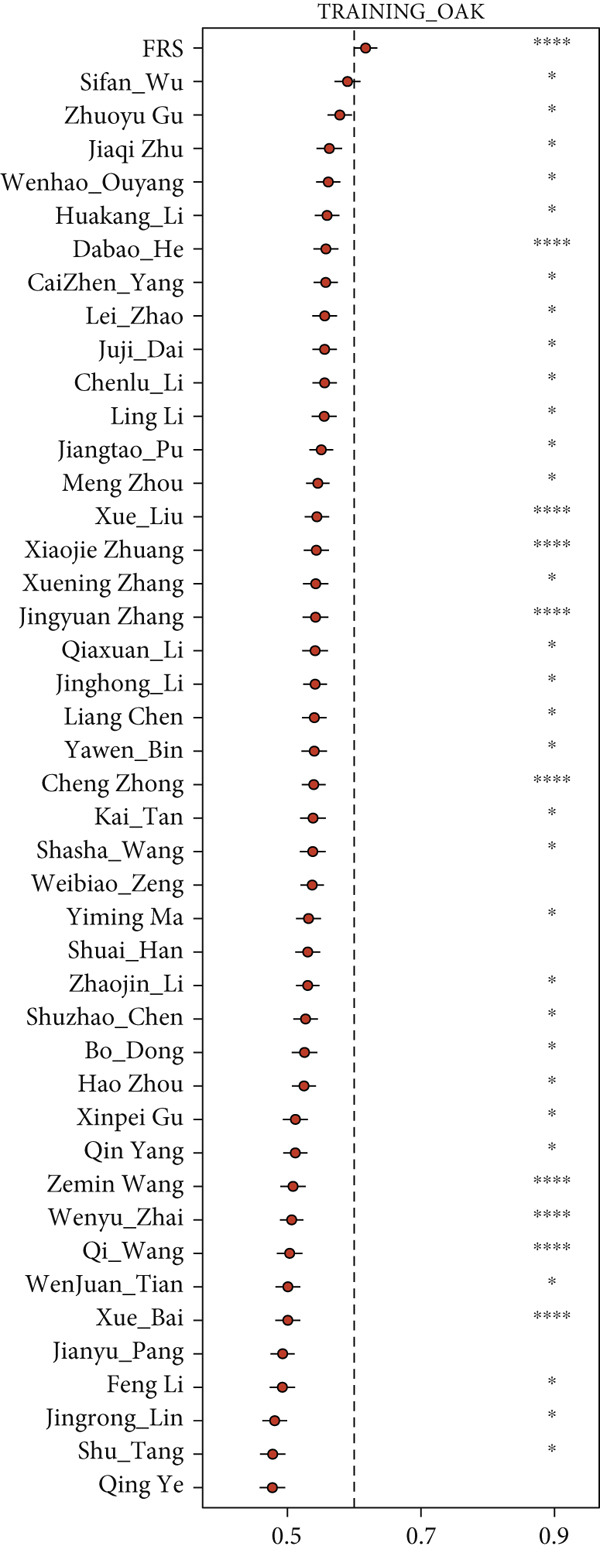
(d)

**Figure:**
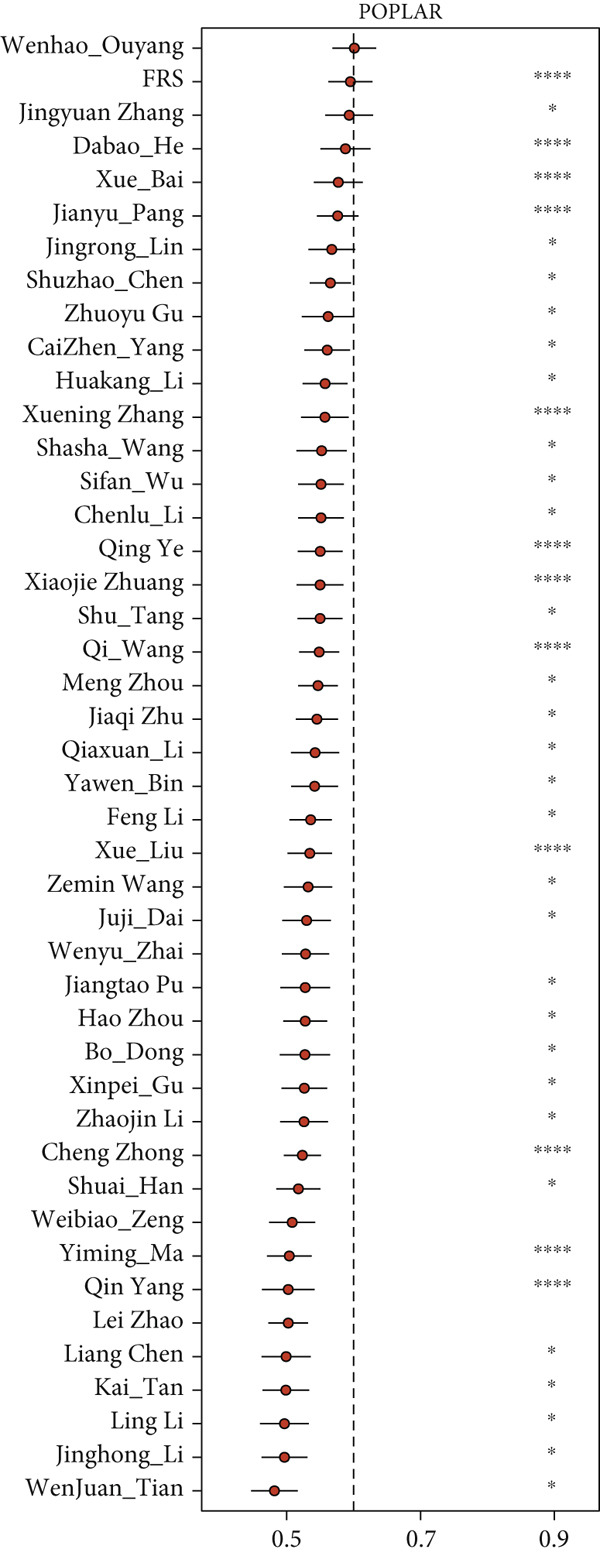
(e)

### 3.4. Biological Mechanisms Underlying FRS

The development of FRS is based on genes characterizing the FOXO‐mediated transcription pathway. FOXOs essentially control antitumor immune responses as well as immune cell homeostasis and development. Therefore, we hypothesized that the high‐risk group and the low‐risk group would differ in gene expression levels and immune infiltration levels. To test this hypothesis, we performed GSEA on the MSigDB database. The analysis yielded a compelling enrichment of immune activation pathways in the low FRS group, as ranked by normalized enrichment score (NES). These pathways encompassed various aspects of immune function, including T cell receptor signaling, antigen binding, interferon‐gamma response, B cell‐mediated immunity, immune effector processes, and immune cell activation and differentiation (Figure 6a). The overrepresentation of these pathways suggests a heightened state of both adaptive and innate immune responses in the low‐risk group. This immunologically active microenvironment may contribute to improved antitumor immunity and favorable clinical outcomes in patients with low FRS scores. This finding suggests a close correlation between FRS and tumor prognosis, which has significant implications for elucidating the pathogenesis of tumors and developing tailored treatment strategies.

Figure 6Gene set enrichment and immune infiltration analysis of the FRS. (a) Enrichment plot of immune activation pathways in the low FRS group, as ranked by normalized enrichment score (NES). The overrepresentation of these pathways suggests a heightened state of immune responses in the low‐FRS group. (b) Immune infiltration analysis based on the CIBERSORT algorithm. The majority of immune cells exhibited a significant negative correlation with FRS in both the OAK and meta cohorts.

**Figure:**
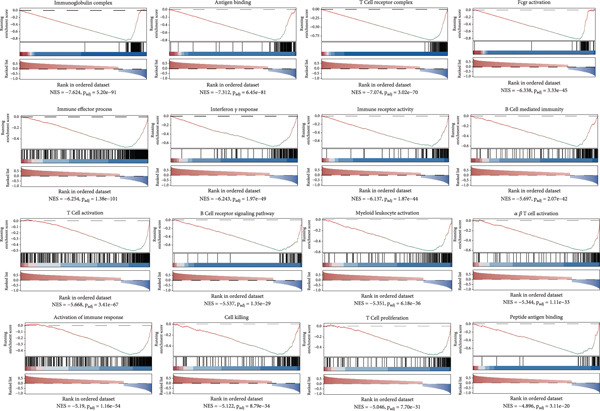
(a)

**Figure:**
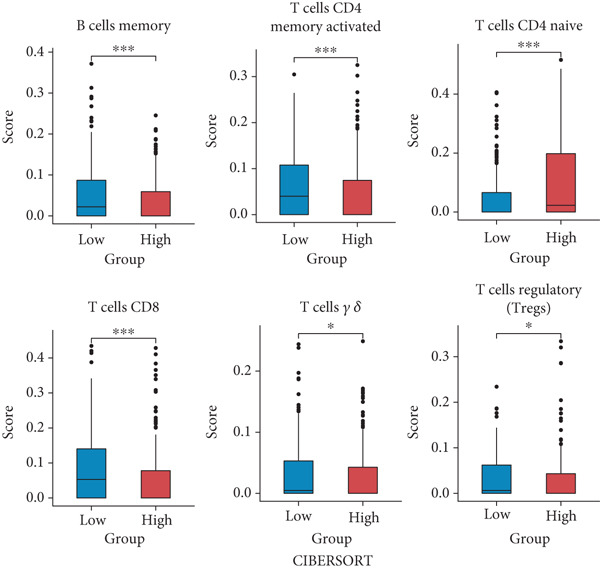
(b)

To assess the differences in the immune cell infiltration status of patients at different FRS levels, we characterized the immune infiltration status of 22 immune cells in NSCLC patients based on the CIBERSORT algorithm. A total of 12 of the 22 immune cells exhibited significant differences between the high‐ and low‐risk groups (Supporting Information 2: Figure S2C). Cellular infiltration analysis demonstrated that the majority of immune cells exhibited a significant negative correlation with FRS in both the OAK and meta cohorts, with a higher abundance of infiltration observed in the low‐risk group (Figure 6b). For instance, the low‐risk group exhibited higher levels of CD8+ T, CD4+ T, gamma delta T cells, neutrophils, macrophages, mast cells, and memory B cells compared to the high‐risk group. Subsequently, we used the xCell algorithm (Supporting Information 2: Figure S2D) to further explore the differences in immune infiltration status between different risk populations. The analysis revealed similar abundance differences. Overall, our multidimensional analysis revealed a significant negative correlation between FRS and immune infiltration abundance.

Furthermore, the predictive performance of FRS in immunotherapy response was validated. The results demonstrated that patients in the low‐risk group exhibited higher objective remission rates. Subsequently, the stromal and immune scores were calculated (Supporting Information 2: Figure S2B) with the ESTIMATE algorithm. The results indicated a positive correlation between FRS and both stromal scores and immune scores, thereby validating the previous conclusions. Concomitantly, an analysis of immune checkpoint correlation demonstrated that a low FRS score was associated with elevated expression of genes such as CTLA4, BTLA, and CD27 and reduced expression of CD276 and VTCN1 genes (Supporting Information 2: Figure S2A).

## 4. Discussion

The incidence of NSCLC is increasing annually, and despite significant advances in standard treatments, patients with NSCLC are frequently diagnosed at an advanced stage, resulting in a poor prognosis. With the advent of molecular biology and immunology, treatment options for NSCLC have expanded, including therapies targeting epidermal growth factor receptor (EGFR) [46]and vascular endothelial growth factor (VEGF) receptors and their ligands [47–49], as well as ICI therapy. The availability of diverse treatment options necessitates the development of more effective personalized assessment methods to inform clinical decisions.

PFS was selected as our primary endpoint due to its ability to directly measure treatment‐specific efficacy without confounding effects from subsequent therapies. PFS provides a clean biological signal for our predictive models by isolating the response to the intervention being studied. This methodological choice enhances model accuracy and reliability while requiring shorter follow‐up periods, allowing for more efficient model development and validation. The PFS endpoint generates timely predictions that can meaningfully influence clinical decision‐making, making it particularly suitable for translational bioinformatics applications where rapid feedback between molecular features and treatment outcomes is essential. However, there is a lack of reliable biomarkers that can consistently identify recurrence‐free survival in “high‐risk” NSCLC patients. To address this gap, we conducted a comprehensive investigation into the relationship between gene transcriptome profiles, recurrence, and immunotherapy.

In this study, a total of 101 models were applied to the training dataset based on four independent cohorts. Further analyses of three independent validation sets demonstrated that the FOXO‐mediated transcription pathway combined with the Lasso + RSF algorithm yielded the highest average *C*‐index for the combined model. The combination of algorithms based on the characterized pathway can further reduce the dimensionality of the variables, making the model simpler and more feasible. Furthermore, ROC and survival analyses demonstrated that FRS maintained stable performance across four independent datasets and metacohorts, suggesting that FRS has strong potential for clinical application. Further evaluation showed that FRS could clearly differentiate between responders and nonresponders to PD‐1 inhibitors, such as atezolizumab and nivolumab. Consequently, FRS may be regarded as a potential biomarker for evaluating the therapeutic efficacy of ICI. Patients with high‐risk FRS may be less suitable for treatment with PD‐1 inhibitors. Moreover, IHC also confirmed the significant correlation between FRS‐related PCK1 and IGFBP1 genes and clinical efficacy, indicating that the FRS model shows promise for use in clinical practice.

The performance of the FRS model, as measured by the *C*‐index, varies across different cohorts, with sample size differences potentially being a key factor [50, 51]. Small sample cohorts, such as Jung, may amplify the model′s randomness, leading to an overestimation of the index. Moreover, patient characteristic heterogeneity, including the distribution of clinical stages (e.g., the proportion of advanced‐stage patients), PD‐L1 expression levels, prior treatment history, and molecular subtypes (e.g., EGFR mutation frequency) across cohorts, directly influences the baseline response to immunotherapy [52, 53]. From a technical platform perspective, differences in RNA sequencing batches (e.g., library preparation methods and sequencing depth), sample sources, and batch correction effects among the cohorts may introduce technical noise [54, 55]. Taking the Jung dataset as an example, Jung has the highest average *C*‐index among the four datasets, and its uniqueness may lie in its smallest sample size (*n* = 27) and single‐center design [26] (the data source for the Jung cohort is highly centralized, with all samples collected from Samsung Medical Center in South Korea; in contrast, the OAK, Poplar, and Ravi cohorts are typical multicenter studies [23–25]). Additionally, the Jung cohort is predominantly composed of Asian populations, while the OAK, Poplar, and Ravi cohorts mainly include European and American populations. This difference may influence the *C*‐index performance through mechanisms such as genetic background and immune microenvironment heterogeneity [56, 57]. Therefore, the high C‐index of Jung should be interpreted with caution, as it may reflect the specific population or technical advantages rather than the universal model performance.

Some traditional clinical features, such as gender [58–60], age [61–64], smoking status [65, 66], and clinical stage [67, 68], are significant in the prognostic assessment and clinical management of NSCLC. Notably, our FRS model not only demonstrated independent predictive performance but also showed excellent accuracy in assessing patients′ prognostic risk based on *C*‐index. Furthermore, we retrieved 43 published immunotherapy predictive models containing multiple biological functions, which we compared to FRS. FRS demonstrated relatively superior performance in each cohort. Certain models performed well in their own training datasets and in some datasets but performed poorly in other datasets, such as Wenhao_Ouyang, possibly due to model overfitting. The FRS model demonstrated superior and more robust predictive performance than all other models in almost all datasets, suggesting that it may be a promising tool for assessing the prognostic risk of NSCLC patients in clinical practice.

FRS quantifies patient risk, but translating this into a clinical tool requires a robust cutoff value [69]. In this study, we employed cohort‐specific thresholds primarily as a validation strategy to demonstrate the FRS′s intrinsic prognostic power across heterogeneous datasets. While this confirms the model′s biological validity, we recognize that a universal threshold is essential for broad clinical application [70].

To address this, we propose a dual pathway for implementation. For near‐term use, a pragmatic approach involves local calibration, where individual institutions establish their own threshold based on a well‐characterized internal patient cohort. This ensures immediate applicability and accuracy within a specific clinical context. Concurrently, the long‐term goal is to establish a single, universal cutoff through validation in a large, multicenter prospective study. This definitive threshold, ideally supported by a standardized assay kit, would ensure consistency and comparability across different healthcare settings, solidifying FRS as a gold‐standard biomarker [71]. This dual strategy offers both a practical solution for early adoption and a clear roadmap toward widespread adoption.

It is of paramount importance to target FRS patients with different levels of FRS for precise treatment. Building on this stratification approach, we investigated the molecular underpinnings of FRS to better understand its biological significance and therapeutic implications. Indeed, FOXO proteins have been demonstrated to play pivotal roles in various cellular and biological functions in cancer, including apoptosis, cancer cell metabolism, cell cycle blockade, and oxidative stress [72–77]. Recently, the capacity of FOXO proteins to regulate the tumor immune response, as well as the homeostasis and development of immune cells, such as T cells, B cells, natural killer (NK) cells, macrophages, and dendritic cells, has been elucidated [78–80]. Consequently, our FRS model, constructed through the FOXO‐mediated transcription pathway, significantly affects the immune status of patients with different levels of FRS, including T cells, antigen‐presenting cells, NK cells, and Treg cells. The results demonstrated that low‐risk FRS patients were predominantly associated with immune activation, which was consistent with their prognostic outcome. Furthermore, such patients exhibited significantly elevated expression of CTLA4, BTLA, CD27, and LAG3 immune checkpoint genes, which supported the conclusion that these patients might benefit more from current immunotherapy. Subsequently, we also explored the underlying biological mechanisms of FRS in depth. The low‐risk group was mainly associated with immune activation. Among the high‐risk FRS patients, there was an enrichment of oncogene methylation‐related pathways, which could explain their high recurrence rate and lower abundance of immune infiltration. This comprehensive understanding of FOXO‐mediated immune regulation and its correlation with treatment outcomes provides a mechanistic foundation for targeting specific molecular pathways in future therapeutic interventions. The robust predictive performance of the FRS across diverse patient populations suggests its potential utility as a companion diagnostic tool to guide the rational design of combination therapies that could enhance immunotherapy efficacy in high‐risk patients [81, 82].

Although FRS is a promising integrative biomarker, it is important to acknowledge the limitations of the model. Firstly, the training and validation datasets for the model were primarily derived from European and American populations, with limited representation from other ethnic groups. Given that the molecular characteristics [83, 84] of NSCLC (such as the prevalence of driver mutations like EGFR), TMEs [85, 57], and response to immunotherapy [86, 87] may vary significantly across different races and geographical populations, the applicability and predictive accuracy of the FRS model in non‐European and non‐American ethnic groups have not been sufficiently validated. Therefore, independent validation in more representative target populations is urgently needed. Secondly, the current study did not assess the model′s generalizability across different treatment regimens or disease stages, which limits the application value of FRS in broader clinical scenarios. Thirdly, the missing data on some clinical and molecular features in the public dataset may have masked potential associations between FRS and some variables. Furthermore, FRS was constructed based on the FOXO‐mediated transcription pathway. However, the ability of FOXO proteins to predict immunotherapy and recurrence‐free survival in NSCLC remains unknown. Finally, all datasets used in this study were retrospective in nature, necessitating prospective comparative studies to rigorously evaluate the FRS model against other emerging clinical and molecular predictive models for NSCLC immunotherapy. Such investigations would confirm the model′s practical utility and reliability in real‐world clinical applications.

## 5. Conclusion

In this study, we integrated multicohort transcriptomic data from 584 NSCLC patients and applied 101 machine learning algorithm combinations across 12,025 pathways. We identified the FOXO‐mediated transcription pathway with the Lasso‐RSF algorithm as the optimal predictor to establish the FRS. The FRS consistently stratified patients into high‐ and low‐risk groups across four independent cohorts and a metacohort, with high‐risk patients showing significantly worse PFS (*p* < 0.05). The signature outperformed 43 published models in predictive accuracy. Biological validation revealed that low‐risk patients exhibited enriched T cell/B cell activation pathways and higher immune infiltration, while IHC confirmed elevated protein expression of FRS core genes (PCK1/IGFBP1) in PD samples. Clinically, FRS serves as a novel pathway‐level biomarker for NSCLC immunotherapy, identifying patients likely to benefit from ICI therapy and guiding treatment strategies, thus advancing precision oncology.

**Nomenclature**
NSCLC: non–small cell lung cancer
ICIs: immune checkpoint inhibitors
RSF: random survival forest
Lasso: least absolute shrinkage and selection operator
CoxBoost: Cox proportional hazards model with component‐wise likelihood‐based boosting
*C*‐index: concordance index
ROC: receiver operating characteristic
GSEA: gene set enrichment analysis
IHC: immunohistochemistry
FRS: FOXO‐related signature
PFS: progression‐free survival
OS: overall survival
PD‐1: programmed death 1
PD‐L1: programmed death ligand 1
TMB: tumor mutation burden
MSI: microsatellite instability
TME: tumor microenvironment
TPM: transcripts per million
Enet: elastic net
GBM: gradient boosting machine
plsRcox: partial least squares regression for Cox models
StepCox: stepwise Cox proportional hazards regression
SuperPC: supervised principal components
survival‐SVM: support vector machine for survival analysis
TNF: tumor necrosis factor
FDR: false discovery rate
logFC: log2 fold change
BSA: bovine serum albumin
FOVs: fields of view
*H*‐score: histochemical score
AUC: area under the curve
PR: partial response
PD: progressive disease
ERS: endoplasmic reticulum stress
NES: normalized enrichment score
EGFR: epidermal growth factor receptor
VEGF: vascular endothelial growth factor
NK: natural killer

## Ethics Statement

The retrospective collection of FFPE samples from NSCLC patients who received ICI therapy for immunohistochemical analysis was approved by the Medical Ethics Committee of Zhujiang Hospital, Southern Medical University (Approval Number 2022‐KY‐208‐01).

## Consent

The authors have nothing to report.

## Disclosure

All authors read and approved the final manuscript.

## Conflicts of Interest

The authors declare no conflicts of interest.

## Author Contributions

Shuqi Wu: writing—original draft, conceptualization, methodology, validation, data curation, visualization. Chenxi Deng: writing—review and editing, formal analysis, data curation, visualization. Chaofan Fan and Qiheng Liang: methodology, investigation, visualization, writing—review and editing. Lingxuan Zhu and Weiming Mou: writing—review and editing, conceptualization, methodology, validation. Hongsen Huang, Keren Wu, Yizhang Li, and Gengwen Deng: visualization, writing—review and editing. Liling Xu, Jiarui Xie, Chenglin Hong, and Yuhang Deng: writing—review and editing, formal analysis. Xingjian Li, Changda Wu, Tao Yang, Peng Luo, and Hank Z. H. Wong: data curation, writing—review and editing. Aimin Jiang, Anqi Lin, and Xin Chen: conceptualization, literature review, project administration, supervision, resources, writing—review and editing. Shuqi Wu, Chenxi Deng, Chaofan Fan, and Qiheng Liang have contributed equally to this work and share first authorship.

## Funding

This work was supported by grants from the Guangdong S&T Program (Grant No. 2023A1111030002) and the Chronic Disease Management Research Project of National Health Commission Capacity Building and Continuing Education Center (Grant No. GWJJMB202510023022).

## Supporting Information

Additional supporting information can be found online in the Supporting Information section.

## Supporting information


**Supporting Information 1** Figure S1. Prognostic analysis of FRS‐related genes in independent datasets. (A–C) Forest plots showing the univariate Cox analysis of FRS‐related genes performed in the (A) Ravi, (B) Jung, and (C) Poplar datasets. The plots show hazard ratios and 95% confidence intervals for genes significantly associated with progression‐free survival.

**Supporting Information 2** Figure S2. Comprehensive immune landscape characterization based on FRS. (A) Correlation analysis of FRS scores and expression levels of immune checkpoint–related genes. (B) Stromal and immune scores calculated based on the ESTIMATE algorithm. (C, D) Immune infiltration status in different risk populations based on CIBERSORT (C) and xCell (D) algorithms.

## Data Availability

The data that support the findings of this study are available from the following sources: The dataset for the Ravi cohort is available in the Database of Genotypes and Phenotypes (dbGaP) under Accession ID phs002822. The raw data for the Jung cohort can be found in the Gene Expression Omnibus (GEO) database with Accession ID GSE135222. The raw sequencing data for the OAK and Poplar cohorts are under restricted access, provided by Genentech. Access to these data can be requested by contacting the provider at devsci-dac-d@gene.com.
