# Supplementary figures and images for: Systematic Pathway Screening via Integrated Machine Learning Identifies FOXO‐Mediated Transcription Signature for Robust Immunotherapy Response Prediction in Non–Small Cell Lung Cancer
Source: Hum Mutat. 2026 Jan 19;2026:8690530. doi: 10.1155/humu/8690530 (PMC12813880; doi:10.1155/humu/8690530)

**A**

**RAVI**

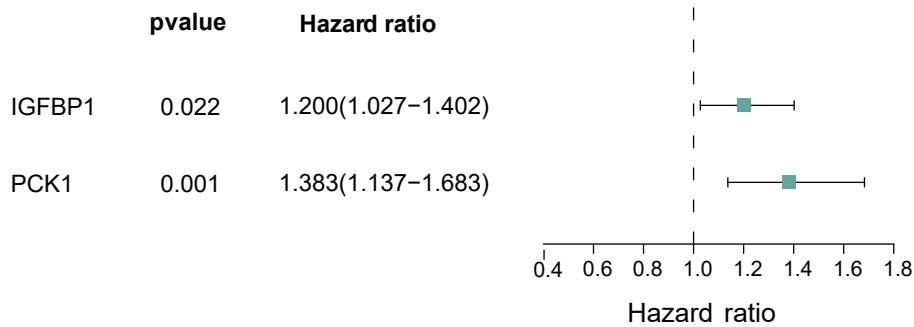

**B**

**JUNG**

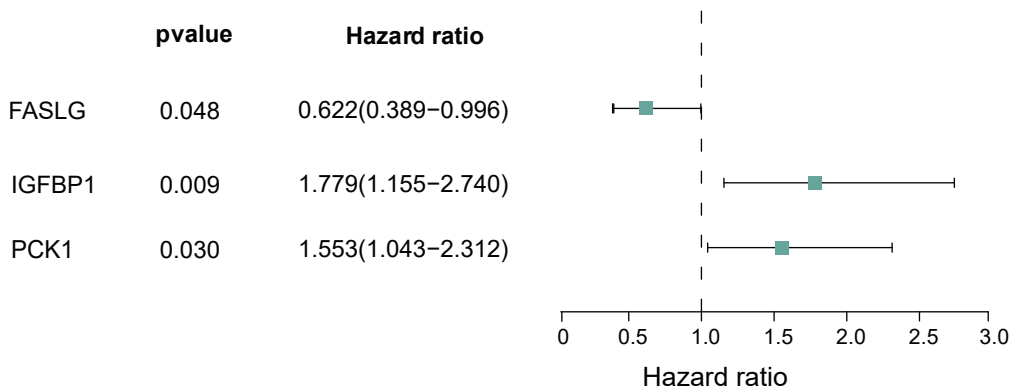

**C**

**POPLAR**

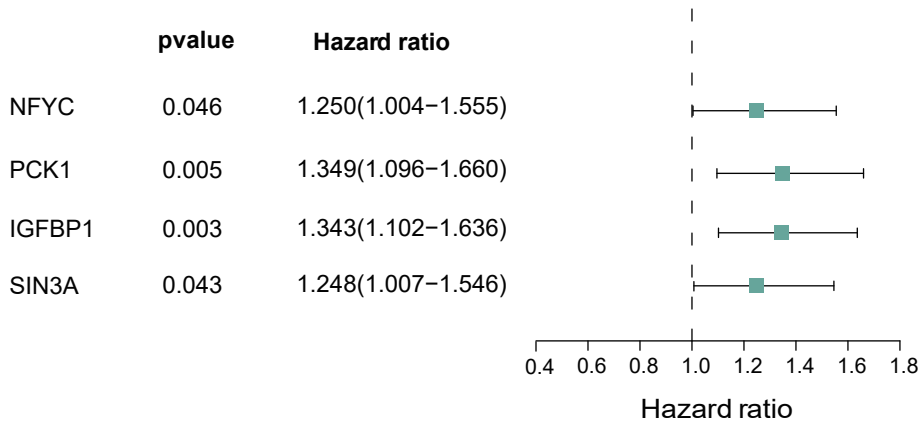

Supplement: Supplementary file 1 — Supporting Information 1 Figure S1. Prognostic analysis of FRS‐related genes in independent datasets. (A–C) Forest plots showing the univariate Cox analysis of FRS‐related genes performed in the (A) Ravi, (B) Jung, and (C) Poplar datasets. The plots show hazard ratios and 95% confidence intervals for genes significantly associated with progression‐free survival. [file HUMU-2026-8690530-s002.pdf]

**A**

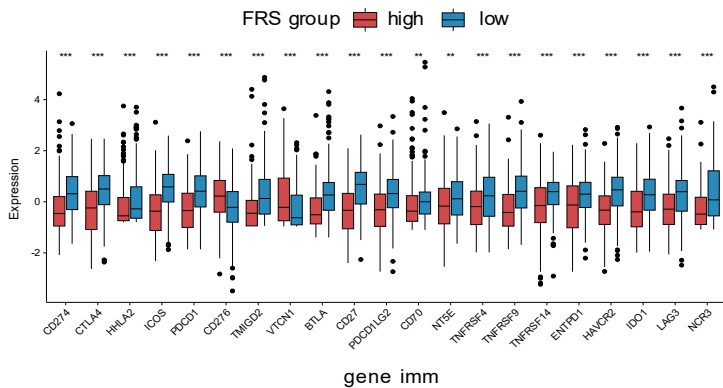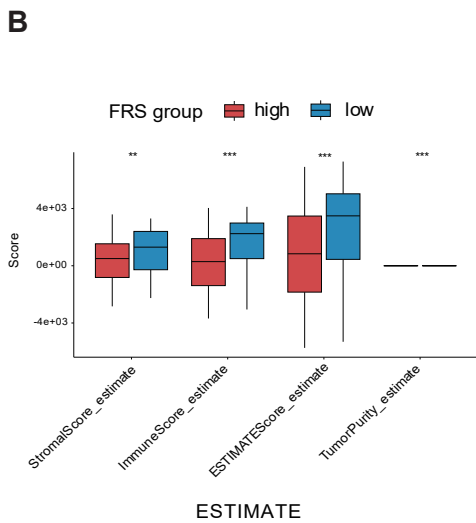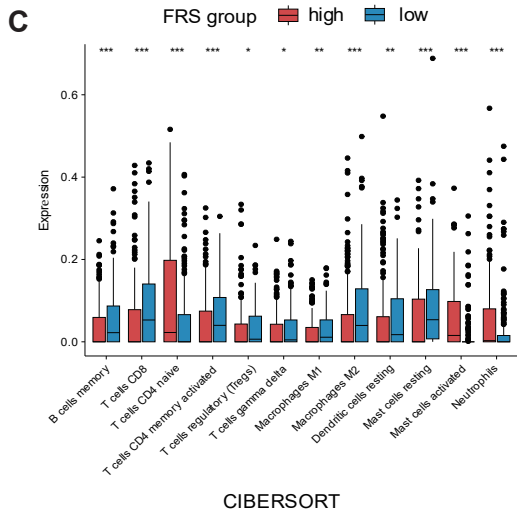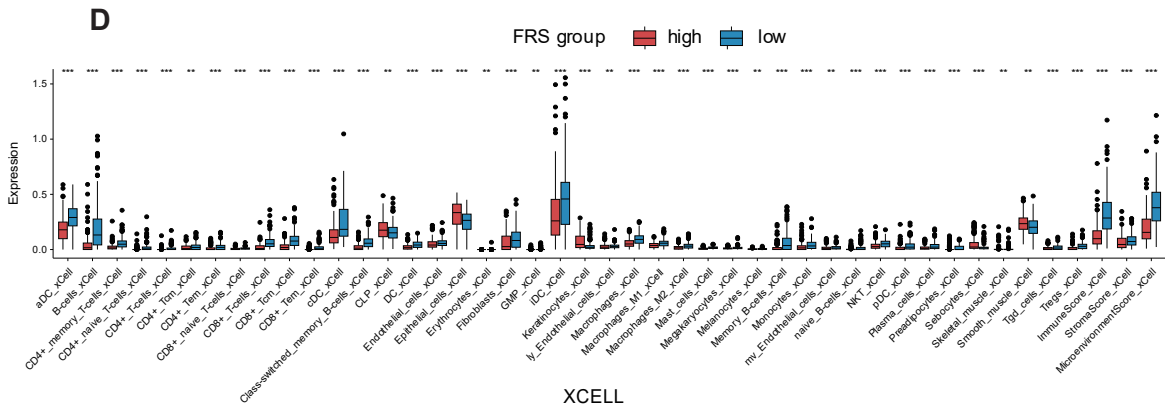

Supplement: Supplementary file 2 — Supporting Information 2 Figure S2. Comprehensive immune landscape characterization based on FRS. (A) Correlation analysis of FRS scores and expression levels of immune checkpoint–related genes. (B) Stromal and immune scores calculated based on the ESTIMATE algorithm. (C, D) Immune infiltration status in different risk populations based on CIBERSORT (C) and xCell (D) algorithms. [file HUMU-2026-8690530-s001.pdf]
